# Choice of satellite imagery and attribution of changes to disturbance type strongly affects forest carbon balance estimates

**DOI:** 10.1186/s13021-015-0041-6

**Published:** 2015-12-15

**Authors:** Vanessa S. Mascorro, Nicholas C. Coops, Werner A. Kurz, Marcela Olguín

**Affiliations:** 1 Faculty of Forestry, Department of Forest Resources Management, University of British Columbia, 2424 Main Mall, Vancouver, BC V6T 1Z4 Canada; 2 Canadian Forest Service, Natural Resources Canada, 506 West Burnside Road, Victoria, BC V8Z 1M5 Canada; 3 Proyecto Mexico-Noruega, Comision Nacional Forestal, Coyoacán, D.F. Col. del Carmen Coyoacán, CP 04100 Mexico, Mexico

**Keywords:** Carbon modeling, CBM-CFS3, Forest disturbance, MRV, Mexico, REDD+, Activity data

## Abstract

**Background:**

Remote sensing products can provide regular and consistent observations of the Earth´s surface to monitor and understand the condition and change of forest ecosystems and to inform estimates of terrestrial carbon dynamics. Yet, challenges remain to select the appropriate satellite data source for ecosystem carbon monitoring. In this study we examine the impacts of three attributes of four remote sensing products derived from Landsat, Landsat-SPOT, and MODIS satellite imagery on estimates of greenhouse gas emissions and removals: (1) the spatial resolution (30 vs. 250 m), (2) the temporal resolution (annual vs. multi-year observations), and (3) the attribution of forest cover changes to disturbance types using supplementary data.

**Results:**

With a spatially-explicit version of the Carbon Budget Model of the Canadian Forest Sector (CBM-CFS3), we produced annual estimates of carbon fluxes from 2002 to 2010 over a 3.2 million ha forested region in the Yucatan Peninsula, Mexico. The cumulative carbon balance for the 9-year period differed by 30.7 million MgC (112.5 million Mg CO_2e_) among the four remote sensing products used. The cumulative difference between scenarios with and without attribution of disturbance types was over 5 million Mg C for a single Landsat scene.

**Conclusions:**

Uncertainty arising from activity data (rates of land-cover changes) can be reduced by, in order of priority, increasing spatial resolution from 250 to 30 m, obtaining annual observations of forest disturbances, and by attributing land-cover changes by disturbance type. Even missing a single year in the land-cover observations can lead to substantial errors in ecosystems with rapid forest regrowth, such as the Yucatan Peninsula.

## Background

Monitoring forest cover and change is essential to quantify the amount of carbon that is stored in the vegetation and soils, and the corresponding greenhouse gas (GHG) emissions and removals [1, 2]. Forest ecosystems can mitigate the impacts of climate change by absorbing significant amounts of atmospheric carbon through plant photosynthesis [3, 4]. However, degradation of forests and conversion to non-forested lands is the second largest anthropogenic cause of carbon dioxide (CO_2_) emissions into the atmosphere [5–8] which has led to initiatives such as Reducing Emissions from Deforestation and Forest Degradation (REDD+) [9].

Natural and anthropogenic disturbances are one of the main drivers that alter the forest structure over time, and understanding their impacts is therefore critical for quantifying carbon stock changes and associated emissions into and removals from the atmosphere [10–13]. Disturbances can range from fire, hurricane, insects and diseases, to timber harvesting, settlement expansion and other human activities. Since each disturbance type impacts the terrestrial carbon cycle in unique ways, annual observations of forest changes by disturbance type are necessary to accurately estimate carbon dynamics in the year of disturbance and the years after the disturbance [5, 10, 14].

Given the complexity of measuring and monitoring the terrestrial carbon cycle, a number of approaches have been developed [7]. Some techniques utilize data collected from ground-plot measurements and allometric equations that relate the physical attributes of trees to aboveground biomass in order to quantify forest carbon stocks (e.g., [15–17]). Other approaches use carbon budget models (e.g., [18]), remote sensing data (e.g. [19, 20]), or a combination of both with field measurements (e.g., [21–24]). Carbon budget modelling is a well-established approach to estimate carbon fluxes at regional to national-scales by integrating data from different spatial and temporal scales [25]. For example, Canada’s National Forest Carbon Monitoring Accounting and Reporting System (NFCMARS) uses the Carbon Budget Model of the Canadian Forest Sector (CBM-CFS3) as the framework that integrates data from many sources [22, 26]. The CBM-CFS3 is also used as a decision support tool for forests managers to quantify the ecosystem carbon dynamics at the landscape level.

In compliance with the Intergovernmental Panel on Climate Change (IPCC) guidelines, the CBM-CFS3 quantifies carbon transfers among the five terrestrial carbon pools: above and belowground biomass, litter, dead wood and soil organic carbon, including atmospheric releases of CO_2_ and non-CO_2_ greenhouse gasses and transfers to the forest product sector [10]. The CBM-CFS3 offers the advantage of modeling the extent to which tree biomass and dead organic matter are affected by different disturbance types [27]. Following disturbance, it simulates subsequent forest transitions and successional dynamics and represents vegetation transfers to litter, coarse woody debris and soil organic carbon pools, and the decomposition of these pools. Based on the stage of stand development and ecological characteristics of the forest, the model accounts for growth and mortality using yield curves.

Carbon models, such as CBM-CFS3 require detailed spatio-temporal information about forest dynamics, including forest disturbance events, to accurately simulate carbon transfers, emissions and removals. Remote sensing (RS) is the primary source of land-cover data for forest monitoring due to its ability to monitor the Earth’s surface on a regular and continuous basis, including areas otherwise difficult to access [28–30]. Recently, an increasing number of regional and global-scale RS studies and products have been developed specifically for forest carbon monitoring and REDD+ at different spatial and temporal scales [20, 31–33]. In a global study, Potter et al. [23] integrated large-scale Moderate Resolution Imaging Spectroradiometer (MODIS) satellite observations with the Carnegie Ames Stanford Approach (CASA), predicting a total of 0.51 Pg C year^−1^ emissions from forest disturbance and biomass burning from 2000 to 2009. At a regional-scale, Masek and Collatz [21] integrated the CASA model with forest inventory data and higher spatial resolution satellite imagery to predict C fluxes from 1973 to 1999. They used Landsat time-series analysis to assess the effects of land-clearing disturbances (such as logging, harvesting and urbanization) on ecosystem productivity and quantified carbon emissions from biomass losses, decomposition and decay.

Significant variability among carbon stock estimates contributes to uncertainty in estimates of emissions and removals. Such variability is potentially due to differences inherent to the methods used, the spatial and temporal scales involved, and the definition of the components included [34]. A recent study by Achard et al. [33] estimated carbon emissions from deforestation in tropical countries for two decades (1990–2000, 2000–2010) by combining forest cover change maps derived from Landsat imagery and pan-tropical biomass maps. Contrasting their results with those obtained by Hansen et al. [35], they found discrepancies that can be explained by different approaches used in the satellite image analysis and their definitions of forest. A global study for tropical countries by Saatchi et al. [31] integrated inventory data and satellite light detection and ranging (LiDAR) data to quantify the carbon stored in the living biomass (247 Pg C) circa 2000. In a similar study, Baccini et al. [32] estimated carbon emissions from pan-tropical deforestation and land-use from 2000 to 2010, combining aboveground biomass with regional deforestation rates derived from LiDAR and MODIS, respectively. Comparing their results with the Forest Resource Assessment (FRA), they found their estimates (228.7 Pg C) were 21 % higher [32]. Likewise, Mitchard et al. [36] compared estimates from Saatchi et al. [31] and Baccini et al. [32] across the Amazon forest to a unique dataset of ground-plot data, and found that the regional differences over or under-estimated the forest carbon estimates obtained from the ground data by >25 %.

It is critical to understand the potential role and implications of different RS observations for estimating ecosystem carbon dynamics following disturbance, in order to reduce uncertainties in measuring and monitoring carbon emissions and removals [37, 38]. In REDD+ studies, land-cover change observations are one of the main components (also called activity data) required for Monitoring Reporting and Verification (MRV) systems [39]. These activity data can be observations of changes in forest areas or land conversions between categories [40]. The design of efficient MRV systems requires an understanding of the implications of different attributes of remote sensing products on estimates of GHG emissions and removals.

This study assesses the impacts of using different RS products on estimates of terrestrial carbon dynamics. We examined the impacts of three attributes of RS products on the estimates of GHG emissions and removals: (1) the spatial resolution (30 vs. 250 m), (2) the temporal resolution (annual observations vs. multi-year observations), and (3) the attribution of forest cover changes to disturbance types. To do so we utilize four contrasting RS datasets—two land-cover change maps and two thematic maps—to compile activity data that we then use as spatially-explicit inputs of annual disturbance events to the CBM-CFS3. The RS data were derived from MODIS, Landsat, and a combination of Landsat-SPOT satellite imagery developed using different approaches, spatial and temporal resolutions. We derived two spatially-explicit layers for each of the RS products, one with land-cover change area, and the second attributing the observed change to its underlying disturbance cause. We then provided these activity data as inputs to CBM-CFS3 and estimated annual and cumulative greenhouse gas fluxes from 2002 to 2010 in the tropical dry forests of the Yucatan Peninsula, Mexico. We conclude with a discussion of key findings and implications of different activity data sources for future carbon budget analysis.

## Methods and data

### Study area

**Fig. 1:**
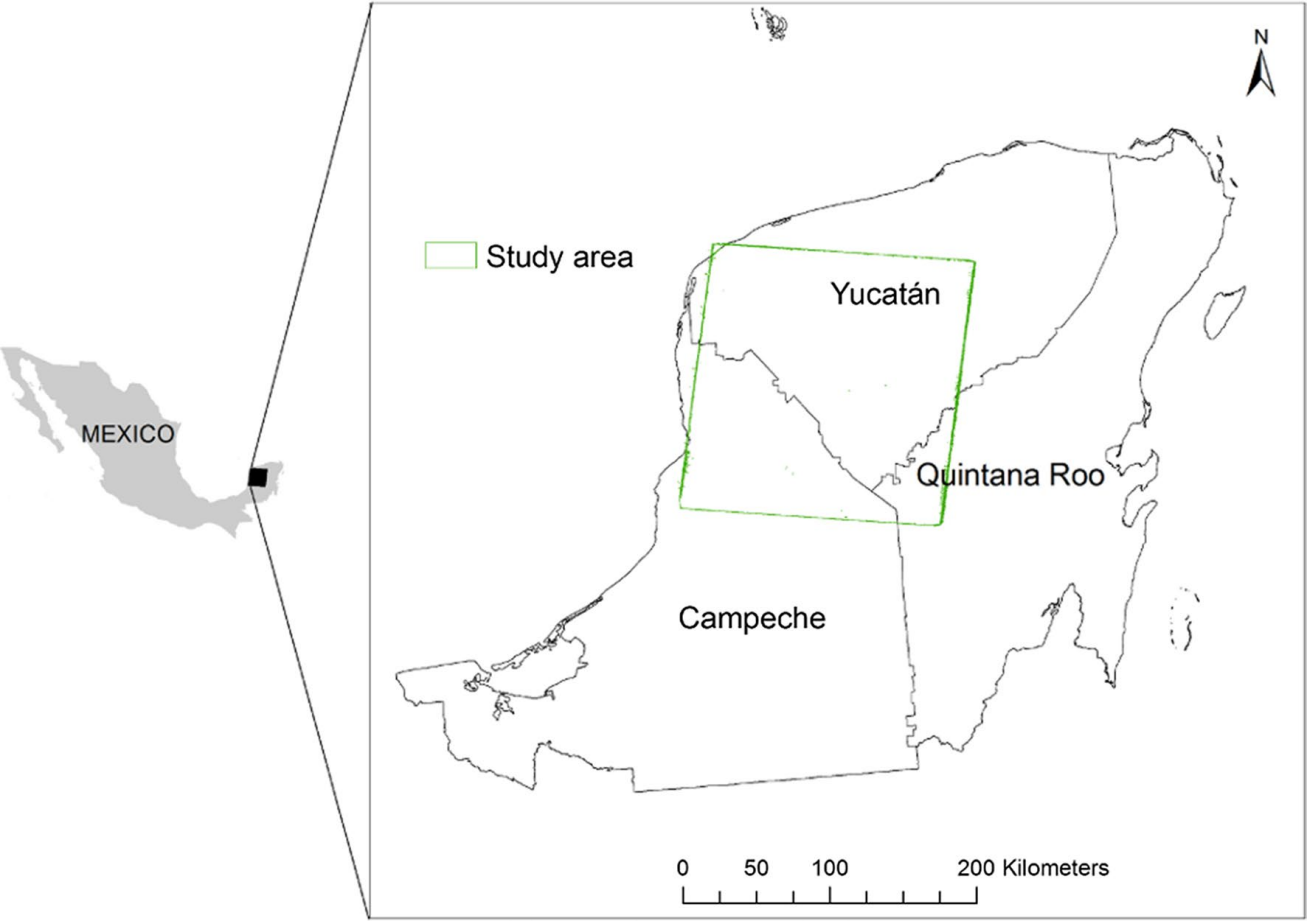
Study area: the Yucatan Peninsula, Mexico

Our study focused on a ~3.2 million ha area located in the northwest side of the Yucatan Peninsula (YP), Mexico, covering parts mainly from Yucatan and Campeche states and a small area of Quintana Roo (see Fig. 1). The region’s vegetation is dominated by secondary lowland dry tropical forests [41] of mixed ages that have regenerated after cycles of shifting agriculture and land abandonment. The region has a tropical climate, with rainy summers from June to October and dry winters from November to May. The mean average temperature is 26 °C with precipitation levels ranging from 900 to 1400 mm year^−1^ [42]. The regional topography is characterized by flat limestone areas and low moderate hills, with an average slope of 7 % and a mean elevation of 116 m [18]. The main soil types are well-drained rendzinas and shallow rocky lithosols [43].

### Remote sensing data

Remote sensing observations are essential to track forest disturbance impacts and changes in land cover and forest carbon at a range of spatial scales [44, 45]. Land-cover changes are those remotely sensed observations of the Earth’s surface that changed from one point in time over two or more time periods because of any disturbance [7], including both natural and human-induced causes. We compiled four remote sensing disturbance products from Landsat, MODIS and a combination of Landsat-SPOT imagery—two classification and two pixel-based maps—derived using a range of approaches to compare their impacts on carbon budget estimates (see Table 1). We use the term pixel-based approach to differentiate the two maps that are labeled pixel by pixel as change/no change, from the thematic or classification maps that are comprised by various pixels grouped together into larger areas sharing similar characteristics (land cover class).

**Table 1:** Remote sensing products used as activity data inputs for carbon modeling with the CBM-CFS3 in the Yucatan Peninsula from 2002 to 2010

Map alias	Source	Type	Satellite	Spatial resolution	Temporality
VCT	NASA	Vegetation Change Tracker map	LANDSAT	30 m	2003, 2004, 2005, 2007, 2008 and 2010
Hansen	University of Maryland	Forest cover loss map	LANDSAT	30 m	2002, 2003… 2010
INEGI	INEGI	Classification map	LANDSAT-SPOT	30 m (25 ha MMU)	2002–2005, 2005–2007, 2007–2011
MODIS	NALCMS/CONABIO	Classification map	MODIS	250 m	2005, 2006… 2011

*MMU* minimum mapping unit

### Landsat: VCT

The vegetation change tracker (VCT) is an automated algorithm that implements a disturbance index (DI, [46]) to reconstruct the history of the landscape disturbance on an annual or bi-annual basis (depending on data quality and availability) using Landsat 30 m time-series analysis [47]. This layer was generated and provided by the NASA Goddard Space Flight Center Biospheric Sciences Research branch. Each pixel of the spatial dataset was labeled according to the year in which the forest cover change was identified, from 1985 to 2010 (represented by 16 classes). From this map, we extracted disturbed areas for the years 2003, 2004, 2005, 2007, 2008 and 2010. Change data were unavailable due to cloud cover in 2002, 2006 and 2009.

### Landsat: Hansen

Annual land-cover change data from a global forest cover change map derived from Landsat imagery at 30 m spatial resolution were also available from Hansen et al. [35]. The coverage provided direct estimates of forest cover loss and gain from 2000 to 2012, including detailed information of annual forest loss. Assessments of forest cover loss included pixels that completely lost tree canopy cover from a stand-replacing disturbance. Here, we obtained annual cover loss areas for our study period (2002–2010).

### Landsat-SPOT: INEGI

We also retrieved three classification maps from the National Institute of Statistics and Geography of Mexico (in Spanish: INEGI): the vegetation and land-use cartography series developed for 2002, 2007 and 2011 at a scale 1:250,000 with approximately 70 classes (INEGI, SIII, SIV, and SV). The vegetation maps were generated using manual interpretation of Landsat and SPOT satellite images, validated with ground-plot data. In addition, a digital elevation model and ancillary datasets that describe the climate, geology, hydrology and topography of the Mexican ecosystems were used in their construction. These maps are a conventional source of information in Mexico for assessing the status and condition of the forested land base. The minimum mapping unit in all three vegetation and land-use cartography series is 25 ha [48]. We used the INEGI maps to generate two land-cover change maps: (1) changes detected from period observation one to two (2002–2007); and 2) changes detected between observation two and three (2007–2011; [49–51]). To simplify this process, we grouped the INEGI vegetation classes into twelve classes established by the National Commission for Knowledge and Use of Biodiversity of Mexico (CONABIO) in the Monitoring Activity Data for the Mexican REDD+ program (MAD-MEX; [48]) and assessed the change from one class to another.

### Modis

Finally, we retrieved six classification maps developed by CONABIO in the North American Land Change Monitoring System (NALCMS) project using MODIS satellite imagery at 250 m spatial resolution [52]. Annual spatially-explicit maps were developed from 2005 to 2011, using monthly composites of MODIS, multiple classifications (ensemble classifier) with decision trees and attributes from auxiliary datasets to characterize 15 land-cover classes in Mexico [53]. These attributes include a digital elevation map, aspect, slope, mean temperature, maximum precipitation, sampling data, and aerial photography at high-spatial resolution. From these maps, we assessed the annual transition from one land-cover class to another from 2005 to 2010.

### Accuracy assessment of forest cover change estimates

We performed an independent assessment of the remote sensing disturbance datasets using ground-plot data obtained from the Mexican National Forest Inventory (NFI). The NFI is the primary data source of information for forest and ecosystem studies in Mexico [54]. Data are collected in a two-stage sampling scheme comprised of primary plots of one-hectare size and four sub-sampling sites of 400 m^2^ each. More than 150 variables are measured on a 5-year cycle, including attributes of the overstory and understory vegetation, the soil, and environmental characteristics of the landscape. For more details concerning the variables, methods and sampling design, readers are referred to the NFI sampling manual [54].We obtained forest change data over the period 2004–2012 of basal area loss/no-loss per hectare computed by Mascorro et al. [55] for 647 plots that were located across the study area. We intersected these plots with the RS derived land-cover change areas to identify and compare the number of plots with forest loss to the proportion of changed areas detected by each RS product using a standard error matrix. The matrix performs a cross-tabulation between the numbers of observations mapped with RS that agree with what is observed on the ground to assess the level of accuracy of the mapped products [56, 57].

### Disturbance attribution

We attributed the land-cover change estimates obtained from the RS products using a Multi-Scale, Multi-Source Disturbance (MS-D) approach developed by Mascorro et al. [55]. This approach integrates RS data, forest inventory and ancillary datasets to attribute the land-cover change observations to the most likely disturbance type (natural or anthropogenic). Using NFI data and historical records of forest disturbances in tabular format (both considered ground-truth data), Mascorro et al. [55] derived annual spatially-explicit layers of major forest disturbance types, and obtained the forest change observed in the ground-plots. A regression tree analysis was undertaken to identify which of the constraining variables (forest disturbance types) best explained the observed forest loss in the field. Once the most likely cause of change was identified, remote sensing maps were used to obtain pixels with land-cover change and overlaid these with the spatially-explicit layers of disturbances characterized by type. Finally, the change was attributed to the most likely disturbance cause according to the relevance resulting from the regression tree analysis.

We retrieved ancillary forest disturbance layers compiled by Mascorro et al. [55] and overlaid them with the VCT, Hansen, MODIS and INEGI RS observations. The disturbance datasets retrieved included annual, spatially-explicit, natural and anthropogenic disturbances from 2005 to 2010: fires, hurricanes, and forest management areas. To characterize the permanent conversion of forestland to non-forestland caused by settlement, we used road coverages retrieved from INEGI [58]. In addition, land-cover classes that changed to urban areas in the classification maps were attributed as settlement.

### Pre-processing data

To prepare the input data and provide the parameters required by the CBM-CFS3 for each of the simulation runs, we used a software tool called “Recliner” developed and provided by the carbon accounting team of the Canadian Forest Service. The tool facilitates the processing of large volumes of spatially-explicit data and assists users with the preparation of required input data for each simulation. We customized Recliner and matched the RS disturbance types with pre-defined disturbance matrices stored in the CBM-CFS3. Disturbance matrices define the impacts on the carbon pools for each disturbance type and specify the amount of carbon that is transferred from the biomass to the dead organic matter pools or is released into the atmosphere during a particular disturbance type [10, 27]. Disturbance matrices also define the amount of carbon transferred from forest ecosystems to the forest product sector by harvesting activities, and the amount of carbon released to the atmosphere as methane (CH_4_), carbon monoxide (CO) and carbon dioxide (CO_2_). Fire disturbances were matched to “wildfires”, settlement to “deforestation” and harvest to “clear-cut with slash-burn”.

The CBM-CFS3 can simulate stand-replacing disturbance by restoring the stand age to the initiation stage, or simulate partial mortality. “Generic mortality” disturbance matrices ranging from 5 to 95 % of impact are among the default choices [27]. Since the CBM-CFS3 does not contain a specific matrix for hurricanes, we matched these events with a generic disturbance matrix of “10 % mortality”, estimated by dividing the mean basal area loss explained by hurricanes in Mascorro et al. [55] studies, over the mean basal area estimated across the Yucatan Peninsula (1.71 m^2^ha^−1^/15.54 m^2^ha^−1^). With Recliner, we assigned the year of disturbance impact to the corresponding land-cover change detection year. In cases where annual data were not available, we distributed equally the disturbance events among the number of years contained in the period of detection (e.g. VCT disturbances detected in 2007 where assigned in equal proportions to 2006 and 2007, since no observations were available for 2006).

### Carbon budget modeling

We provided annual disturbance events to the CBM-CFS3 to quantify carbon exchange from tree biomass mortality, plant detritus decay, soil organic carbon and forest regrowth after disturbance. Additional parameters and data required by the CBM-CFS3 for the simulations were provided by the carbon accounting team of the Canadian Forest Service, and researchers from the Mexican Forest Service. These parameters were kept constant in all simulations so that only the activity data differed among the eight scenarios.

Ancillary datasets included detailed information on forest inventory, forest type, forest status, ecological boundaries, age-class structure, and yield curves [59]. Stand age and yield curve data, in combination with the ecological characteristics of the site, are used by the CBM-CFS3 to simulate forest growth and carbon accumulation [10]. The stage of stand development is also used to simulate litterfall and decomposition rates for dead organic matter and soil carbon. For forest status, we used protected and non-protected conservations areas, which the model employs as a classifier to differentiate the conditions on the landscape. We also used as a classifier the ecoregions level-I retrieved from the Commission for Environmental Cooperation (CEC) [60]: tropical humid forests and tropical dry forests. Estimates of carbon sinks and sources were then generated with the CBM-CFS3 for each of the two spatially-explicit layers of the four RS products, i.e. with and without attribution of cover loss to disturbance types. For simulations without attribution to specific disturbance types we assumed that all forest cover changes were caused by stand-replacing harvest with slash-burning.

## Results

### Forest change detection

**Fig. 2:**
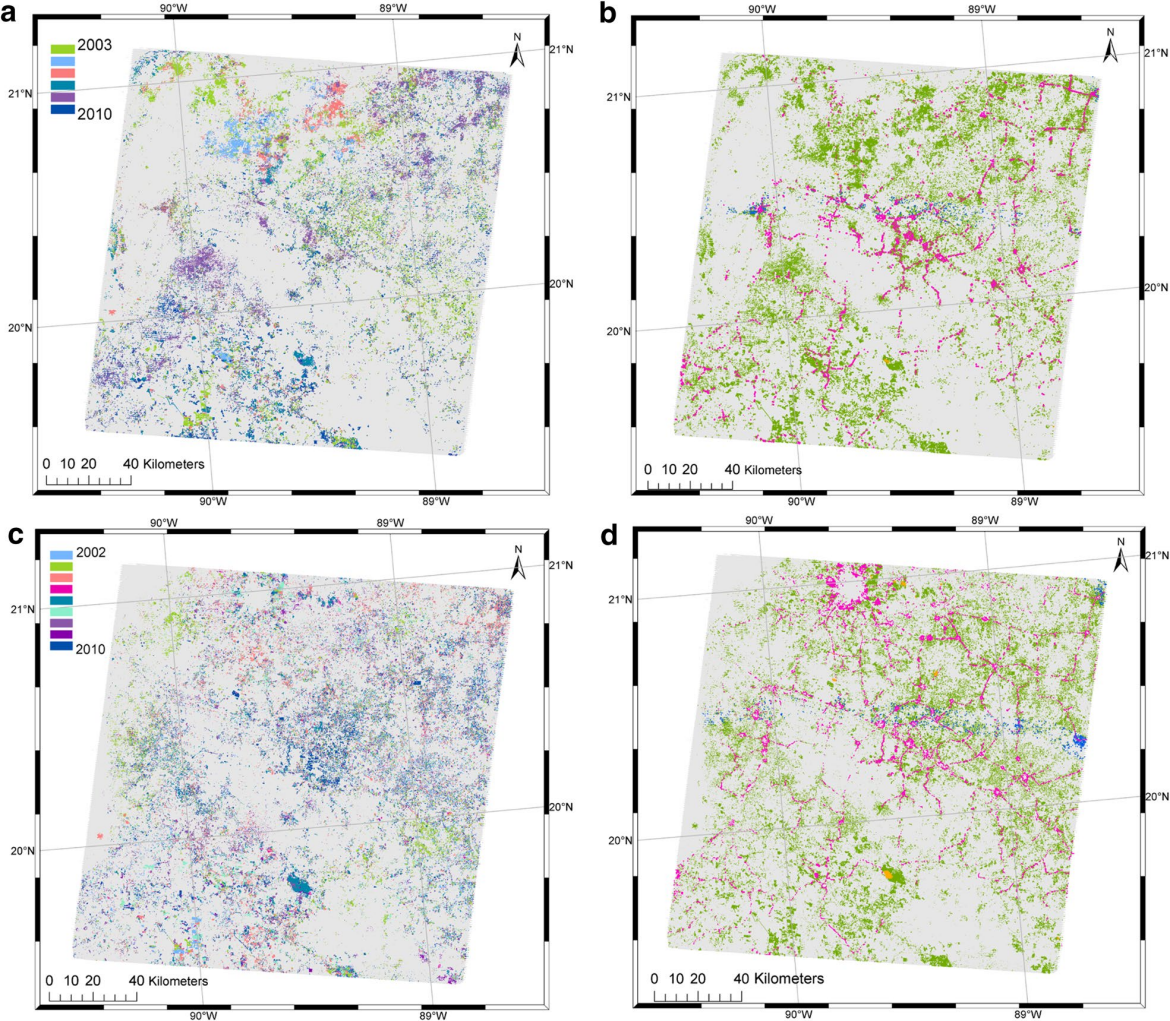
Land-cover change maps derived from the different remote sensing products. VCT map: (**a**, **b**); Hansen map: (**c**, **d**). *Left side* maps show annual non-attributed disturbances; *right side* maps show attributed disturbances: *green* harvest, *pink* settlement, *blue* hurricane, *orange* fire

**Fig. 3:**
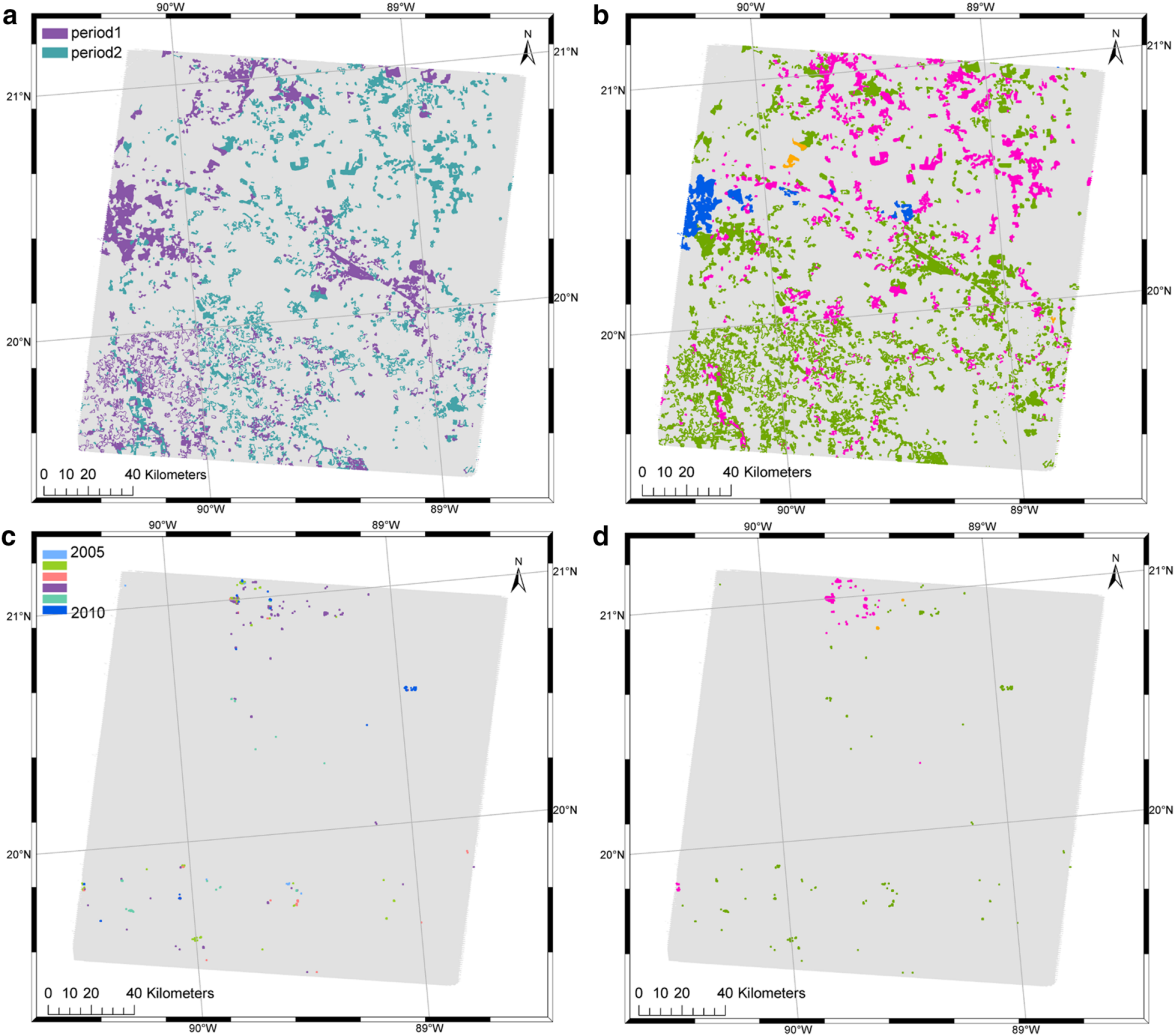
Land-cover change maps derived from the different remote sensing products (thematic). INEGI maps: (**a**, **b**); and the MODIS maps: (**c**, **d**). *Left side* maps show annual non-attributed disturbances; *right side* maps show attributed disturbances: *green* harvest, *pink* settlement, *blue* hurricane, *orange* fire

The spatial variation in the location and extent of changes in forest area detected by the VCT, Hansen, MODIS and INEGI maps can be observed in Figs. 2 and 3. Overall, MODIS products detected much less changes across the landscape than the Landsat products, likely due to its lower spatial resolution. As can be seen in Fig. 2b, d, landscape changes between the VCT and Hansen products exhibited similar patterns. With a 30 m spatial resolution, these products detected finer disturbance events throughout the study area. Undisturbed forest areas were primarily located in the center of the region, expanding to the northwest and southeast of the area. In contrast, the INEGI maps generated with a classification approach detected larger contiguous areas of change from land-cover class transitions, due to the various 25 ha MMU’s of similar characteristics grouped together into a class (Fig. 3e, f).

**Fig. 4:**
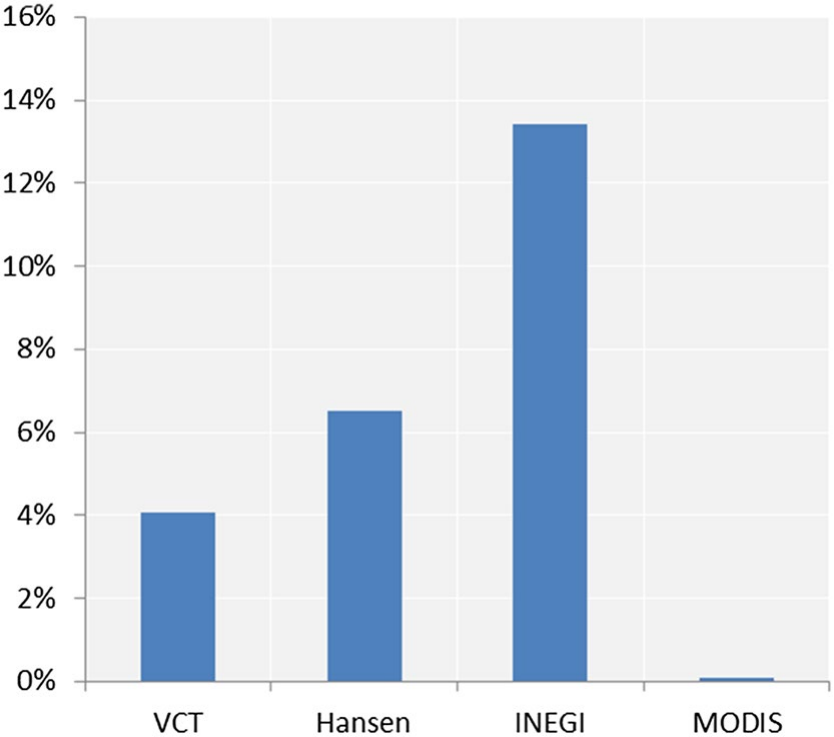
Cumulative area with change (as percentage of total area) detected in the study area from 2002 to 2010 with Landsat (VCT, Hansen and INEGI) and MODIS satellite imagery

The cumulative area of change detected in the study area by each RS product across the analysis period is shown in Fig. 4. It is apparent that the Landsat products detected the most change in all of the remote sensing approaches. However, the amount detected was less in the pixel-based approach than that derived from the classification maps (INEGI).

**Fig. 5:**
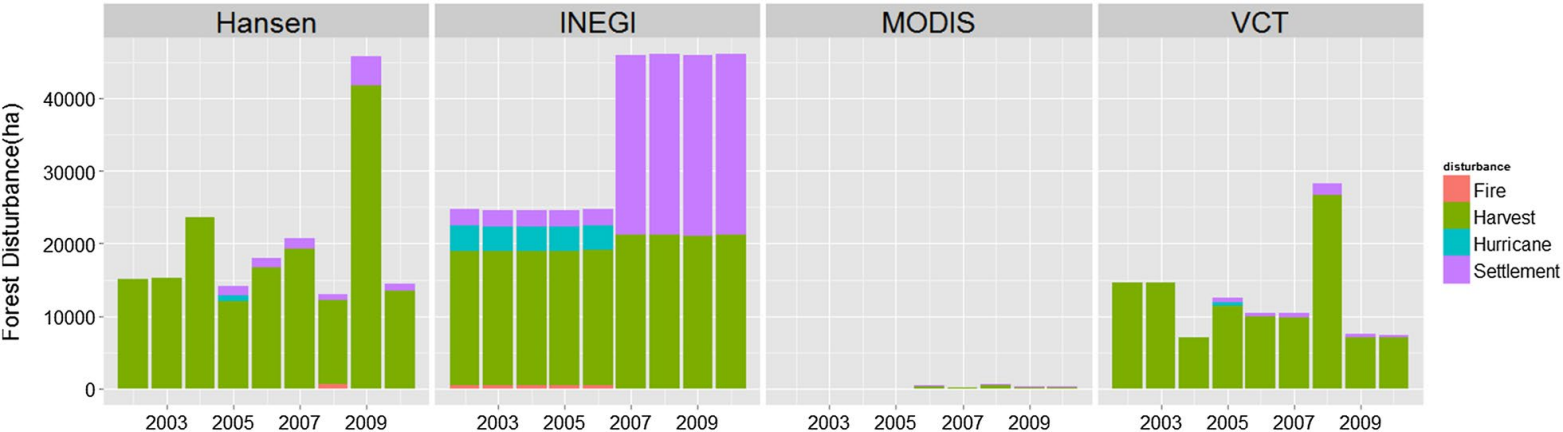
Annual area changed by disturbance type derived from Landsat (VCT, Hansen and INEGI) and MODIS remote sensing land-cover change products

The range between the maximum (13 % by INEGI) and minimum (1 % by MODIS) cumulative percentages of area change detected, highlights the uncertainties resulting from the differences in spatial and temporal characteristics inherent in each RS product. Temporal trajectories of the land-cover changes detected in annual time-steps provided additional information on the inter-annual variation of disturbance events as shown in Fig. 5.

### Forest change accuracy assessment

Table 2, summarizes the results of intersecting the NFI plots describing forest cover loss, with the VCT, Hansen, and INEGI land-cover change products in an error matrix. Of the 647 NFI plots located in the study area, 119 showed a forest cover loss, and 528 had no evidence of any basal area reduction in the plot. The first row of each table contains the number of mapping units that each RS product detected as forest cover loss compared to what was observed on the ground with the NFI plots (the total sum of the first column). As can be seen in Table 2a and b, the overall accuracy of the non-classification maps, the VCT (83 %) and Hansen (82 %), maps produced using pixel-based image processing approaches was similar, whereas the INEGI accuracy was 79 % (Table 2c). However, we can observe that all three maps presented a low producer’s accuracy, detecting only a small fraction of the changes observed in the NFI plots likely due to forest degradation. The largest uncertainties were found in the MODIS maps, as there was no correspondence between the MODIS changed pixels and the NFI plots.

**Table 2:** Error matrix of landsat maps VCT (a), Hansen (b) and INEGI (c) identification of change and no change areas compared to the ground-plot data

(a) VCT				
	Change	No change	Sum	Producer’s
NFI plots				
Change	18	101	119	15 %
No change	11	517	528	98 %
Sum	29	618	647	
User’s	62 %	84 %		
Overall accuracy = 83 %				

(b) Hansen				
	Change	No change	Sum	Producer’s
NFI plots				
Change	27	92	119	23 %
No change	22	506	528	96 %
Sum	49	598	647	
User’s	55 %	85 %		
Overall accuracy = 82 %				

(c) INEGI				
	Change	No change	Sum	Producer’s
NFI plots				
Change	26	93	119	22 %
No change	44	484	528	92 %
Sum	70	577	647	
User’s	37 %	84 %		
Overall accuracy = 79 %				

### The impact of activity data on estimates of carbon fluxes

**Fig. 6:**
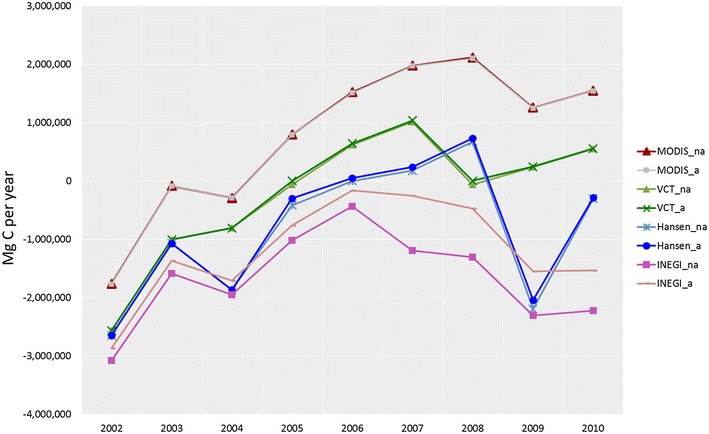
Annual carbon fluxes estimated with different sources of activity data in the Yucatan Peninsula from 2002 to 2010; *a* attributed, *na* no-attributed

We used the CBM-CFS3 model to simulate the effects of activity data on emission and removal estimates during, and following disturbances from 2002 to 2010 (Fig. 6). Results show the annual net changes in ecosystem carbon stocks, with negative numbers indicating a decrease in carbon stocks (an emission of carbon to the atmosphere) and positive numbers an increase in carbon stocks (carbon uptake from the atmosphere). The estimates show both differences in the magnitude of changes and differences in trends due to differences in activity data.

The values from these simulations should not be considered as absolute values as research continues to improve yield curves and other ecological and modelling parameters that will likely affect future estimates of fluxes. What matters here is the impact of the activity data on the differences in the annual and cumulative estimates of carbon fluxes (Fig. 6).

**Fig. 7:**
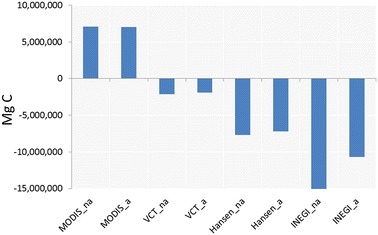
Cumulative carbon fluxes, estimated with different sources of activity data in the Yucatan Peninsula from 2002 to 2010; *a* attributed, *na* no-attributed

The cumulative difference in the carbon balance estimates resulting from the four RS products for the 9-year period (2002–2010) was over 30.7 million Mg C (112.5 million Mg CO_2e_) see Fig. 7, with MODIS-derived estimates suggesting a large sink while INEGI-derived estimates remained a source for the whole period (Fig. 6).

Since the ecological parameters and additional data inputs used in each of the simulations were kept constant, the observed differences in the estimates reflect only the effects of the spatial and temporal resolutions inherent to each of the RS observations, and the impacts of attributing cover changes to disturbance types with different impacts on carbon stocks.

Over the simulation period we observe the overriding trend of the MODIS disturbance detection underestimating cover changes and the associated carbon losses compared to the Landsat RS products. MODIS-based simulations shifted from a source to a sink in 2005 and remained a sink for the remainder of the simulations. Landsat-derived estimates in the other hand, followed a similar trend in 2002 and 2003, and began diverging in 2004. From 2005 to 2007, simulations based on VCT and Hansen predicted increasing carbon stocks. Consistent with the amount of land-cover change detected by each product (Fig. 5), the rate of increase in carbon uptake estimated with VCT from 2005 to 2007 was higher than that simulated with Hansen. In 2007, Hansen removals were 234,188 Mg C, whereas the VCT estimates were 1,033,608 Mg C (Fig. 6). While some disturbance events were detected in 2005–2007, the amount of area affected was not enough to bring the sink back to a source (e.g., estimates in 2009 from Hansen). Fewer disturbances were observed by VCT in this period, which translated to fewer carbon emissions and more carbon accumulation due to forest growth without disturbance. Nevertheless, in 2008 estimates from these two data sources went in opposite directions.

In the Hansen simulation, the forest continued to sequester carbon in 2008 as the amount of affected area decreased by 37 % compared to 2007 (Fig. 5). This was translated by the CBM-CFS as less carbon emissions and more living biomass accumulation, sequestering 3 times more carbon than in 2007 (234,188 Mg C in 2007 vs 729,215 Mg C in 2008). Conversely, with the VCT, the amount of change area detected in 2008 was more than double compared to 2007 (10,409 vs 28,278 ha), releasing considerable amounts of carbon (1,033,964 Mg C) to the atmosphere from forest disturbance events that killed the vegetation and redistributed the carbon in the dead organic matter pools during that year.

Moreover, Mascorro et al. [55] found that 2009 was an intense year of disturbances in the Yucatan Peninsula. Accordingly, the impact of the disturbance events detected by Hansen, resulted in significant amounts of carbon emissions into the atmosphere (stocks dropped by 2,774,453 Mg C; Fig. 6). MODIS-derived estimates also showed a decrease in carbon content in 2009, but by much smaller amount due to its limited capacity to detect changes in the landscape. However, with the VCT no land-cover change observations were included in 2009 due to limitations of image acquisition and cloud coverage, and therefore the simulations show no decrease in carbon emissions in 2009, despite the fact that the change observed in 2010 was equally distributed to 2009 and 2010. It is likely that some of the 2009 disturbance events were not detected by the VCT in 2010 due to the fast regrowth rate of the tropical forests of the Yucatan Peninsula.

**Fig. 8:**
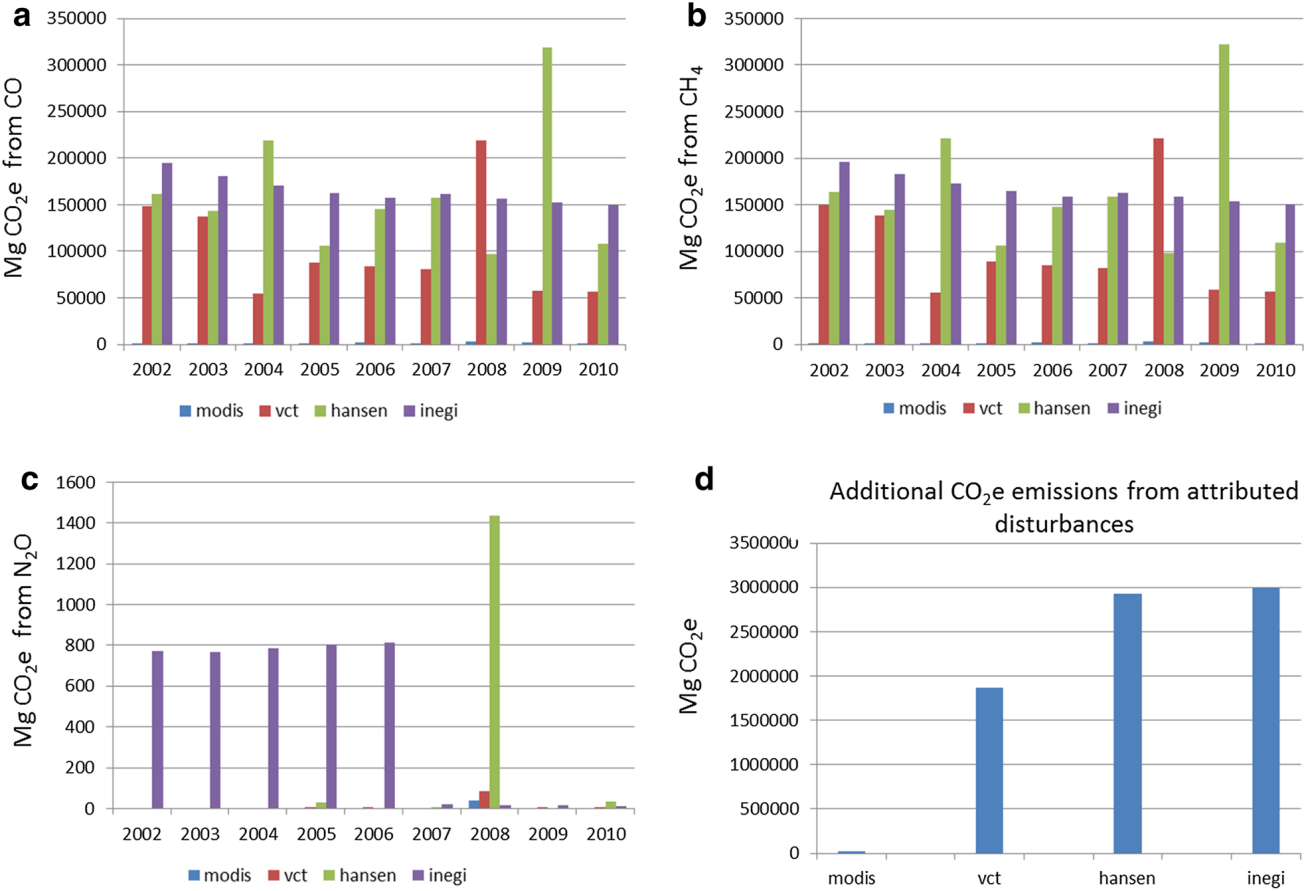
CO_2_ equivalent GHG emissions from carbon monoxide CO (**a**), methane (CH_4_) (**b**) and nitrous oxide N_2_O (**c**) with a Global Warming Potential (GWP) time horizon of 100 years, and their cumulative-additional CO_2_e emissions from attributed disturbance types (**d**)

Overall, estimates from non-attributed changes presented more carbon emissions than the attributed estimates in all the simulations. In the INEGI simulations this difference was more evident. In the second period, emissions from the non-attributed disturbances (7 million Mg C) were more than double than the attributed ones (3.2 million Mg C). When converting the additional non-CO_2_ GHG emissions from fires (see Fig. 8a–c) in the form of carbon monoxide (CO), methane (CH_4_) and nitrous oxide (N_2_O) with their global warming potential (GWP) to 100 years (1, 25 and 298 respectively), we can see the additional CO_2_ equivalent (CO_2_e) emissions contributed by non-CO_2_ greenhouse gases (i.e. 3 million Mg CO_2_e more; see Fig. 8d).

## Discussion

This study analyzed the contribution of three main attributes from RS activity data on estimates of forest carbon dynamics: (1) the spatial resolution (30 vs. 250 m), (2) the temporal resolution (annual vs. multi-year observations), and (3) the attribution of forest cover changes to disturbance types. Annual carbon fluxes were simulated with the CBM-CFS3 from 2002 to 2010 over a 180 × 180 km study area of dry tropical forests in the Yucatan Peninsula, Mexico.

### Spatial resolution

The large difference in annual and cumulative carbon estimates generated with MODIS and Landsat-derived disturbance observations suggests that increased spatial resolution should be the first priority when deriving activity data for carbon modeling. Carbon flux estimates derived from Landsat at higher spatial resolution reflected the impacts of more accurate activity data. In contrast, the coarse spatial resolution of MODIS-derived products results in a substantial underestimate of change events and thus overestimates forest carbon sinks over the observation period. The latter occurred despite having MODIS observations on an annual basis from 2005 to 2010. While Mascorro et al. [55] were able to characterize major stand-replacing disturbances for the entire Yucatan Peninsula with MODIS, results from this study showed that this 250 m resolution satellite imagery has limitations for providing activity data for carbon modeling at finer spatial scales. Typically, satellite imagery from MODIS has focused on broad scale studies (e.g., [23, 29]).

Moreover, we found that the pixel-based image processing approach using VCT and Hansen was more accurate than change inferred from classification maps (i.e., INEGI, MODIS) (see Table 2). While the INEGI simulations of the first period observations (2002–2006) show a similar trend to VCT and Hansen, change observations for the period 2007–2011 are much higher than any of the others. This is likely due to the fact that INEGI maps were generated with a classification approach, detecting larger areas of change formed by clusters of pixels that share similar characteristics and attributes. Therefore the larger disturbances observed over the second period, resulted in higher estimates of change likely due to larger areas that transitioned from one class to another (as opposed to single-pixel changes). Some of the predicted changes using thematic maps can also result from inaccuracies inherent to the classification method [61, 62], and omission and commission errors [63]. Nevertheless, post-classification analysis—a change detection technique that compares the initial and final class condition between two classification maps in time- has become a popular method for change detection [64], that helped to better identify settlement expansion embedded in the land-cover classification approach as urban areas. Yet, RS products generated with a pixel-based approach presented some limitations in attributing the changes to the underlying disturbance type due to the lack of spatially-explicit ancillary datasets. Further research would be required to increase the accuracy of land-cover changes characterized by disturbance type using additional datasets.

### Temporal resolution

Reliable estimates of carbon dynamics require detailed observations of drivers of change on annual basis [14]. Our results showed that higher temporal resolution RS products improved the carbon dynamics estimates and captured the inter-annual variability in the forest. Consistent with the large disturbances detected in 2009, carbon fluxes predicted with the Hansen and MODIS products showed a decrease in total ecosystem carbon content. However, due to the lack of disturbance events detected with the VCT in 2009, corresponding decreases in the carbon stocks from the disturbance events were not reflected in the CBM-CFS3 estimates. This occurred despite the fact that the disturbance events observed over the period 2009–2010 were equally distributed in 2009 and 2010. This result showed that even missing a single year in the land-cover observations can lead to substantial errors; especially in ecosystems with rapid-regrowth forests, such as the Yucatan Peninsula.

### Attribution of land-cover changes by disturbance type

Natural and human-induced disturbances modify the stand age and succession dynamics of the landscape in a unique way, changing the live biomass and dead organic matter stocks and subsequent turnover [65, 66]. Results from this study showed the relevance of identifying the cause of change in the landscape. The cumulative difference between scenarios with and without attribution of disturbance types was over 5 million Mg C for a single Landsat scene over the 9-year-period. INEGI estimates over the second period (2007–2011) from the non-attributed disturbances, for example, dropped 84 % more than the attributed ones yielding higher emissions (3.2 million Mg C more). Since the default disturbance type associated with harvesting was clear-cut with slash-burn, it generated more emissions because it involved burning. Depending on the disturbance type, the CBM-CFS3 disturbance matrices determines how much biomass gets killed, re-distributing the carbon in the litter and dead organic matter pools accordingly; affecting the carbon changes more adequately. These differences between with and without attribution were even more pronounced when reported as CO_2_ equivalent emissions (as required in REDD+ projects, see Fig. 8a–c), because fires cause additional non-CO_2_ GHG emissions (in the form of CH_4_ and N_2_O), with higher global warming potentials than CO_2_ (see Fig. 8d).

The carbon balance estimates obtained for this study are based on CBM-CFS3 simulations using preliminary inventory, age distribution and yield data for the study area [59]. Ongoing research as part of bigger collaboration of members from CONAFOR´s project “Reinforcing REDD+ and South–South Cooperation”, SilvaCarbon program, Colegio de Postgraduados, and research supported by the CEC will refine and improve the CBM-CFS3 input data. The absolute values of the numerical estimates obtained in this study may thus change in the future, but the general conclusions about the impacts on carbon balance estimates of the spatial and temporal resolution of RS products, and the attribution of land-cover change to causes of disturbance will not be affected by future changes to other input data of the model.

## Conclusions

Systematic forest monitoring, reporting and verification (MRV) systems are required to aid in the successful development of national and regional strategies for REDD+, and to ensure long-term commitments to preserve forests [25, 40, 67]. This can only be achieved by implementing RS observations that will allow monitoring of large areas of land in a regular, consistent, and cost-efficient way and by developing modelling tools that can translate activity data derived from RS products into policy-relevant estimates of GHG emissions and removals. Results from this study provide an improved understanding of the role of RS disturbance observations on ecosystem carbon dynamics and the range of variability of carbon flux estimates following disturbance. Developing climate change mitigation scenarios, priorities, and initiatives requires further knowledge of the drivers of deforestation and forest degradation that can have a significant impact in the reduction of GHG emissions [7, 25, 68].

We conclude that spatial scale represents a serious mapping constraint when attempting to encompass large forest areas for ecosystem carbon accounting. Therefore, carbon monitoring decisions should consider direct trade-offs between the spatial detail of finer resolution products (e.g., Landsat, LiDAR, RapidEye) with more precision and less area covered per scene, versus moderate and broad spatial resolution observations derived at larger scales (e.g., MODIS). Our results support findings from the Global Forest Observations Initiative (GFOI) regarding MODIS imagery as “too large to be used for generating REDD+ activity data”, suggesting it could be used in complimentary applications (e.g., monitoring near-real time forest change indicators) [69].

This research documents cumulative differences from four different RS products in estimates of carbon emissions of 30.7 million Mg C (112.5 million Mg CO_2_e) over a 9-year period in a single Landsat scene in Mexico. While we did not attempt to extrapolate this to the national-scale implications, it is evident that with 135 Landsat scenes required to cover all of Mexico [48], the choice of satellite data sources to derive activity data can have a large impact on uncertainties in national estimates of forest carbon budgets. The magnitude of these differences highlights that efforts to improve the activity data can yield substantial reductions in uncertainty of GHG estimates at the regional or national scale. Uncertainties arising from activity data can be reduced by, in order of priority, increasing spatial resolution from 250 to 30 m, obtaining annual observations of forest disturbances, and by attributing land-cover changes by disturbance type. Results from this study can help justify the monitoring expenses required to reduce uncertainties in GHG emissions and removals estimates for countries like Mexico, with a variety of small scale-low impact disturbances and a strong interest in participating in REDD+ initiatives. Improved estimates also help meet the requirements of the Intergovernmental Panel on Climate Change (IPCC) to identify, quantify and reduce sources of uncertainty in estimates of GHG emissions and removals as far as is practicable.

## Abbreviations

CEC: Commission for Environmental Cooperation
CBM-CFS3: carbon budget model of the Canadian Forest Sector
CONABIO: National Commission for Knowledge and Use of Biodiversity of Mexico
GHG: greenhouse gas
INEGI: National Institute of Statistics and Geography of Mexico
LiDAR: light detection and ranging
MODIS: moderate resolution imaging spectroradiometer
NFI: Mexican National Forest Inventory
REDD+: reducing emissions from deforestation and forest degradation
RS: remote sensing
VCT: vegetation change tracker algorithm
YP: Yucatan Peninsula
